# Increasing the glucose metabolism enhances the bioelectricity generation in microbial fuel cells: Glucose metabolism enhances the bioelectricity generation

**DOI:** 10.3724/abbs.2022129

**Published:** 2022-09-07

**Authors:** Zhenyu Guo, Lei Wang, Changyuan Yu

**Affiliations:** College of Life Science and Technology Beijing University of Chemical Technology Beijing 100029 China

Electrochemically active bacteria (EAB) is the core component in microbial fuel cell devices, which convert chemical energy into electricity by catalyzing the oxidation of various carbon sources [ 1, 2] . Two logical strategies could be achieved to enhance the electricity producing ability of EABs [3]. One is screening of new kinds of microbials from the environment, the other is genetic modification of current available EABs to optimize their electron producing processes, such as glucose metabolism.

Generally, *Escherichia coli* degrades glucose through the glycolysis pathway, in parallel with the oxidative phosphorylation to produce ATP [ 4, 5] . Phosphofructokinase (PFK) and pyruvate kinase (PK) catalyze two irreversible steps during glycolysis [ 6, 7] . Increasing the concentrations of these two enzymes intracellularly may enhance the metabolism of glucose. As a result, more protons would be released and then be utilized by MFC devices.

Following this hypothesis, recombinant plasmids containing wild-type *PFK1*, *PFK2*, *PK1* and *PK2* genes from *E*. *coli* ( Supplementary Table S1) were constructed, respectively. Gene overexpression in *E*. *coli* BL21(DE3) was induced by 50 mg/mL IPTG for about 8 hrs at 37°C. Bacteria were collected, and the total mRNA and proteins were extracted. Significant increases of the cDNA levels were detected by RT-PCR ( Figure 1A). The overexpressions of proteins were also assessed after SDS-polyacrylamide gel electrophoresis (SDS-PAGE) by Coomassie Brilliant Blue staining ( Figure 1B).

**Figure 1:**
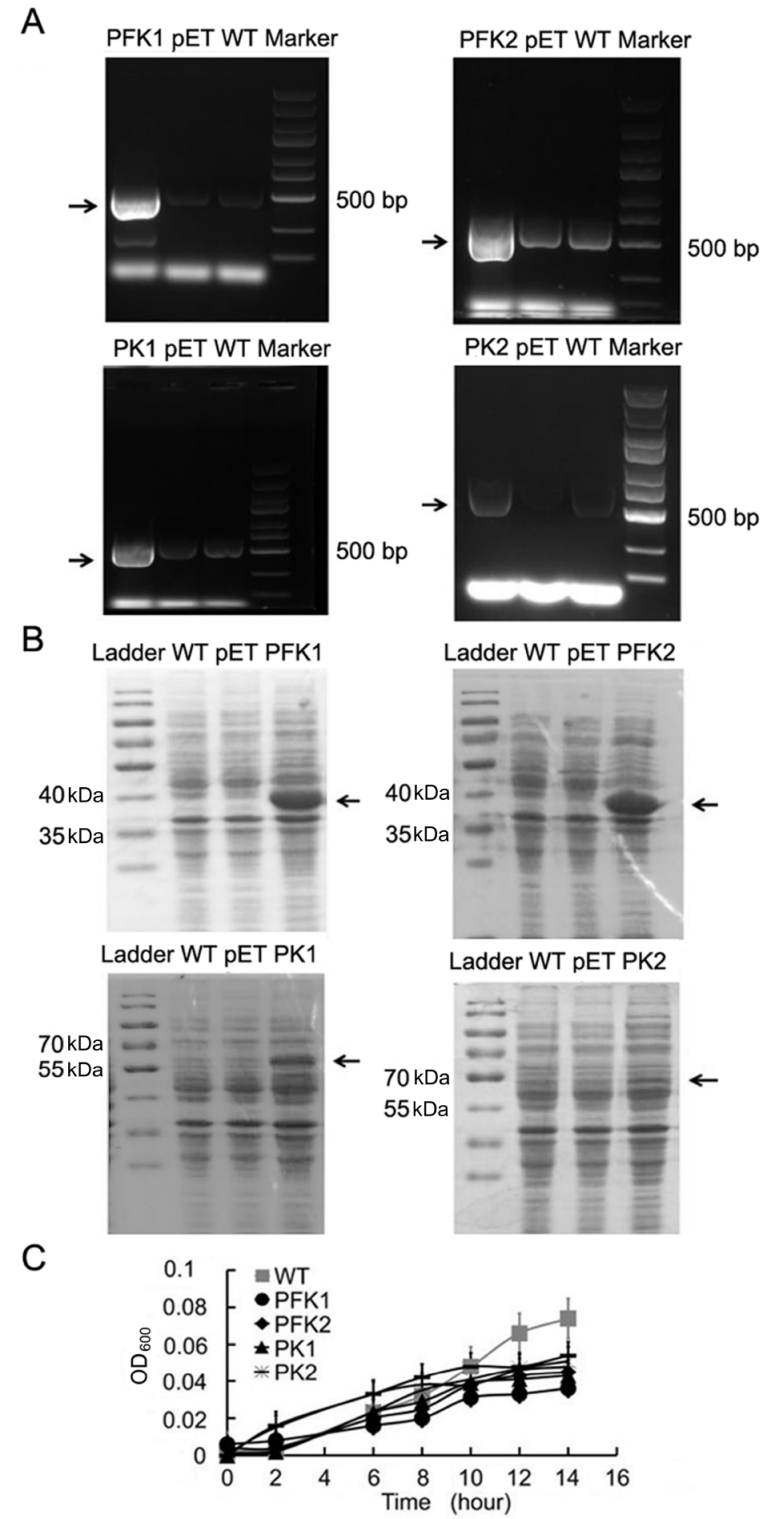
Figure 1 Expressions of recombinant genes in *E*. *coli* BL21 after IPTG induction (A) Representative data of RT-PCR. (B) SDS-PAGE. (C) Growth curves.

Overexpression of exogeneous proteins may influence the growth status of bacteria. We then plotted the growth curves with OD _600_ values of cultured medium with bacteria ( Figure 1C). When cultured in standard LB medium, over-expressions of these four genes all suppressed the growth of *E*. *coli*. However, when cultured in anodic buffer, which used glucose as carbon source, the cell concentrations varied in different groups ( Supplementary Table S2).

To study the bioelectricity-producing ability of recombinant bacteria, we used two-chamber MFCs as reported previously [8]. In this device, anodic and cathodic chambers were physically separated by a proton exchange membrane (Dupont, Beijng, China). Carbon cloth (Qiandingli, Suzhou, China) was utilized as the electrode in each chamber. Voltage outputs were detected by EM9636 voltage collector (Zhongtai, Beijing, China). Two glucose concentrations were used in this study: 5 g/L and 10 g/L. Compared with the wild-type strain, *E*. *coli* with over-expressed genes, under certain conditions, significantly enhanced the electricity-producing efficiency, as indicated in voltage output curves and the averaged peak output values ( Figure 2).

**Figure 2:**
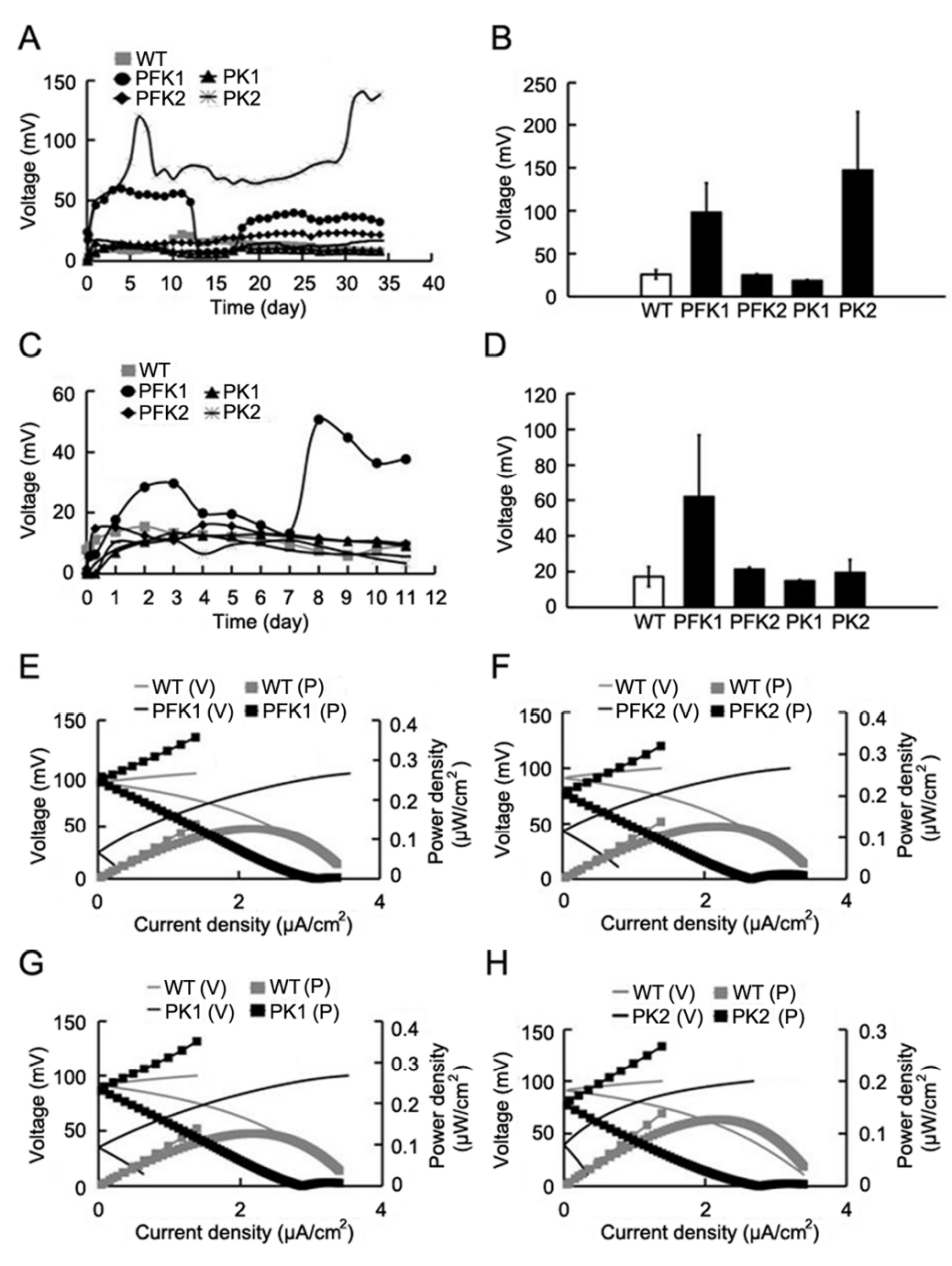
Figure 2 Overexpressions of glycolytic genes enhanced electric energy outputs of MFCs The voltage output curves and the peak values were plotted for MFCs with 5 g/L (A,B) or 10 g/L (C,D) glucose respectively. (E‒H) The overexpressions of four gene recombinant strains increased the power density of MFCs.

To analyze the overall performance of MFCs, we recorded the 7-day power density (10 g/L glucose) with electrochemical workstation (Chenhua, Shanghai, China). As shown in Figure 2, the power densities in PFK1, PFK2, PK1 and PK2 groups were 158.01%, 130.87%, 153.83% and 93.47% of the control group, which indicated a potential enhanced performance.

Furthermore, we measured the COD values of anodic buffer from each group after the voltage recording, in order to determine the consumption efficiency of carbon resources ( Supplementary Table S3). All groups showed higher ratios in glucose utilization at 5 g/L than those in control group while PFK2 and PK2 groups showed higher at 10 g/L.

The performance of MFCs is closely related to the energy metabolism of EABs. Regulation of genes involved in proton and electron transfer could significantly influence the electrochemical ability of EABs. For example, down regulation of *cymA* and *mtr* genes in *Shewanella oneidensis* MR-1 affected the electron transfer and reduced the electrochemical properties [8]. In this study, overexpressing PFK and PK might theoretically increase the glycolysis in *E*. *coli* and enhance the bioelectricity generation. Our data further suggest the effectiveness of genetic modification on EABs to optimize their performances in MFCs.

## Supplementary Data

Supplementary data is available at *Acta Biochimica et Biophysica Sinica* online.

## Supporting information

22192Supplementary_File
