# Supplementary material for: Increasing the glucose metabolism enhances the bioelectricity generation in microbial fuel cells: Glucose metabolism enhances the bioelectricity generation
Source: Acta Biochim Biophys Sin (Shanghai). 2022 Sep 7;54(10):1566–7. doi: 10.3724/abbs.2022129 (PMC9828298; doi:10.3724/abbs.2022129)
Supplement: 22192Supplementary_File [file 22192Supplementary_File.pdf]

Supplementary Materials and Methods

Anode buffer: glucose (5, 10 g/L), ammonium chloride (0.19 g/L), sodium chloride (0.5 g/L), calcium chloride dihydrate (0.03 g/L), magnesium sulfate heptahydrate (0.03 g/L), sodium hydrogen carbonate (1 g/L), potassium phosphate monobasic (5 g/L), potassium phosphate dibasic (3.86 g/L), trace elements (12.5 mL/L).

Cathode buffer: sodium chloride (0.5 g/L), calcium chloride dihydrate (0.03 g/L), magnesium sulfate heptahydrate (0.03 g/L), sodium hydrogen carbonate (1 g/L), potassium phosphate monobasic (5 g/L), potassium phosphate dibasic (3.86 g/L), sodium nitrate (0.3 g/L), trace elements (12.5 mL/L).

Trace elements: nitrilotriacetic acid (1.5 g/L), magnesium sulfate heptahydrate (6.14 g/L), manganous sulfate (0.5 g/L), sodium chloride (1 g/L), iron (II) sulfate heptaphdrate (0.1 g/L), calcium chloride dihydrate (0.1 g/L), cobaltous chloride (0.1 g/L), zinc chloride (0.13 g/L), copper (II) sulfate pentahydrate (0.01 g/L), aluminium potassium sulfate dodecahydrate (0.01 g/L), boric acid (0.01 g/L), sodium molybdate (0.03 g/L).

Data, except the power density, were averaged from 3 independent experiments. Results were expressed as the mean ± SEM. T-test with SPSS 19.0 software was used to perform the statistical analysis. $P<00.5$ was considered as significant difference.

Supplementary Table S1. Sequence of primers used in this study

Primer	Primer sequences (5′→3′)
PFK1Forward	CGCGGATCCATGATTAAGAAAATCGGTGTGTTGACAA
PFK1 Reverse	CCGTCTCGAGATACAGTTTTTTCGCGCAGT
PFK2 Forward	CCGGAATTCATGGTACGTATCTATACGTTGACACTT
PFK2 Reverse	CCGTCTCGAGGCGGGAAAGGTAAGCGTAAATT
PK1 Forward	CGCGGATCCATGAAAAAGACCAAAATTGTTTGCACCATCGGA
PK1 Reverse	CCC**G**AAGCTTCAGGACGTGAACAGATGCGGTGTTAGTA
PK2 Forward	CGCGGATCCATGTCCAGAAGGCTTCGCAGAA
PK2 Reverse	CCGTCTCGAGCTCTACCGTTAAAATACGCGTGGTATTAGTAGAA
PFK1 cDNA Forward	AGACCGTATGGTACAGCTAGACCGTTA
PFK1 cDNA Reverse	AATGCGCCAGTTCGTCAACATCA
PFK2 cDNA Forward	CATTGCCCATCTTGGAGGCAGTGC
PFK2 cDNA Reverse	CTGTCACCAGCGCCAACGGTACT
PK1 cDNA Forward	GTCTGTTGGCAACACCGTACTGGTT
PK1 cDNA Reverse	ATAGAAACCGCTTCCAGCGGGTATTTAC
PK2 cDNA Forward	TCCTGCTCGACGCCAACCTGGGTAAAG
PK2 cDNA Reverse	AGAAACGTTGATGCTCGGGATTTTTTCCGCACC

Supplementary Table S2. Bacterial concentrations under different glucose concentrations

Genotype	OD_{600}	Glucose (g/L)
Wild-type	0.28±0.07	
PFK1	0.31±0.03	
PFK2	0.21±0.05	5
PK1	0.28±0.03	
PK2	0.27±0.04	
Wild-type	0.29±0.02	
PFK1	0.25±0.02	
PFK2	0.22±0.01*	10
PK1	0.24±0.03	
PK2	0.21±0.06	

*P<0.05, compared with wild-type group.

Supplementary Table S3. COD under different glucose concentrations

Genotype	COD (mg/L)	Glucose (g/L)
Wild-type	1623.30±15.90	
PFK1	972.60±271.30	
PFK2	1592.95±14.05	5
PK1	1596.15±18.65	
PK2	1600.10±37.80	
Wild-type	4296.10±209.20	
PFK1	5419.55±430.35	
PFK2	3998.80±1046.60	10
PK1	5901.40±166.70*	
PK2	843.30±3.70**	

**P*<0.05, ***P*<0.01 compared with wild-type group.
